# Riemannian geometry-based metrics to measure and reinforce user performance changes during brain-computer interface user training

**DOI:** 10.3389/fncom.2023.1108889

**Published:** 2023-02-13

**Authors:** Nicolas Ivanov, Tom Chau

**Affiliations:** ^1^PRISM Lab, Bloorview Research Institute, Holland Bloorview Kids Rehabilitation Hospital, Toronto, ON, Canada; ^2^Institute of Biomedical Engineering, University of Toronto, Toronto, ON, Canada

**Keywords:** brain-computer interface (BCI), electroencephalography (EEG), user training, Riemannian geometry, user evaluation, simulation

## Abstract

Despite growing interest and research into brain-computer interfaces (BCI), their usage remains limited outside of research laboratories. One reason for this is BCI inefficiency, the phenomenon where a significant number of potential users are unable to produce machine-discernible brain signal patterns to control the devices. To reduce the prevalence of BCI inefficiency, some have advocated for novel user-training protocols that enable users to more effectively modulate their neural activity. Important considerations for the design of these protocols are the assessment measures that are used for evaluating user performance and for providing feedback that guides skill acquisition. Herein, we present three trial-wise adaptations (running, sliding window and weighted average) of Riemannian geometry-based user-performance metrics (*classDistinct* reflecting the degree of class separability and *classStability* reflecting the level of within-class consistency) to enable feedback to the user following each individual trial. We evaluated these metrics, along with conventional classifier feedback, using simulated and previously recorded sensorimotor rhythm-BCI data to assess their correlation with and discrimination of broader trends in user performance. Analysis revealed that the sliding window and weighted average variants of our proposed trial-wise Riemannian geometry-based metrics more accurately reflected performance changes during BCI sessions compared to conventional classifier output. The results indicate the metrics are a viable method for evaluating and tracking user performance changes during BCI-user training and, therefore, further investigation into how these metrics may be presented to users during training is warranted.

## 1. Introduction

Although brain-computer interfaces (BCI) have been proposed as an access technology for individuals with severe motor impairments (Wolpaw et al., 2002; Neuper et al., 2003), users often struggle to produce consistent and machine-discernible neural patterns, thereby limiting clinical adoption (Lotte et al., 2013; Jeunet et al., 2016a; Lotte and Jeunet, 2018; Sannelli et al., 2019). This inability of classification algorithms to correctly decode user patterns with sufficient accuracy is referred to as BCI inefficiency (Vidaurre and Blankertz, 2010; Vidaurre et al., 2011b; Sannelli et al., 2019) and has been repeatedly observed in BCI studies since their inception (Allison and Neuper, 2010).

While BCI inefficiency has been tackled *via* sophisticated signal processing and classification approaches, only modest improvements to BCI decoding capability have been reported (see Lotte et al., 2018 for review). Inter-user performance differences remain significantly larger than inter-classifier differences within users (e.g., Ang et al., 2012; Barachant et al., 2012; Lawhern et al., 2018; Li et al., 2019) suggesting that BCI inefficiency cannot be addressable strictly by algorithmic enhancements. Indeed, BCI user performance has been associated with a melange of physiological (Blankertz et al., 2010; Ahn et al., 2013a,b; Zhang et al., 2015; Shu et al., 2018) and psychological factors (Burde and Blankertz, 2006; Grosse-Wentrup et al., 2011; Hammer et al., 2012; Witte et al., 2013; Jeunet et al., 2015; Kleih and Kübler, 2015; Myrden and Chau, 2015; Ahn et al., 2018).

Increasingly, BCI task performance is being recognized as a skill that can be learned (Lotte et al., 2013; Ono et al., 2013; Jeunet et al., 2016b; Lotte and Jeunet, 2018; Perdikis et al., 2018; Meng and He, 2019; Nguyen et al., 2019; Benaroch et al., 2021), lending credence to supportive skill development as a meaningful avenue to reduce BCI inefficiency. Indeed, studies have already demonstrated that commonly deployed user training approaches do not promote skill learning in BCI users (Lotte et al., 2013; Jeunet et al., 2016a). Mastery of a BCI control task can be characterized as procedural learning (Kober et al., 2013; Hiremath et al., 2015; Casimo et al., 2017), whereby procedural memory is developed by systematically repeating the task until all the required actions occur automatically, without conscious control (Eichenbaum, 2008). Fitts and Posner (1967) and Anderson (1982) posit that the first stage of such learning entails developing clear knowledge of the task, how it can be performed successfully, and how to identify erroneous or poor task performance. Thus, BCI skill learning hinges on the provision of digestible and accurate extrinsic feedback (Lotte et al., 2013; Jeunet et al., 2016a; Lotte and Jeunet, 2018); humans cannot intrinsically evaluate their brain signals. However, due to the complexities of EEG interpretation and its low signal-to-noise ratio, relatively few metrics have been proposed as user feedback.

Specifically, we contend that BCI user feedback must:

1. Contain, but not be limited to, descriptive information regarding the current level of performance (Lotte et al., 2013; Jeunet et al., 2016a; Lotte and Jeunet, 2018).
2. Reflect performance change such that the learner receives actionable guidance toward incremental improvement (Cannon and Witherspoon, 2005; Hattie and Timperley, 2007; Ghaderi and Farrell, 2020).
3. Be available immediately following task trial performance (200–2,000 ms) as procedural learning relies on the timely association between action performance and reinforcement-induced dopamine (Schultz, 2002; Perrin and Venance, 2019).

Table 1 organizes past research on EEG BCI user performance metrics under two groups, those derived from EEG signals while the user remains at rest, and those that depend on EEG collected during active brain states (e.g., performance of a mental task). However, only one approach meets all three design criteria.

**Table 1:** BCI user performance metrics.

	**References**	**Metric**	**Purpose**	**Criteria**			**Remark**
				**1**	**2**	**3**	
Resting	Blankertz et al. (2010) and Sannelli et al. (2019)	SMR predictor	Predict CA	✓			Not designed for instantaneous feedback; requires recordings of brain at rest
	Ahn et al. (2013b)	(*P*_α_+*P*_β_)/(*P*_θ_+*P*_γ_)	Predict CA	✓			
	Zhang et al. (2015)	M1 spectral entropy	Predict CA	✓			
Active brain state	Wolpaw et al. (2002) and Wolpaw et al. (2000)	Strength of SMR suppression	Feedback	✓		✓	No synthesis of current and Previous feedback
	Pfurtscheller et al. (2003) and Sannelli et al. (2019)	Classifier-based, predicted task label	Feedback			✓	May not reflect changes in user performance; difficult to interpret
	Bamdadian et al. (2014)	Pre-trial onset EEG signal power	Predict CA	✓			Pre-trial activity may not reflect task performance
	Shu et al. (2018)	Laterality index based on mean event-related EEG signal power	Predict CA	✓			Not conducive to online use—needs user-specific frequencies
	Lotte and Jeunet (2018)	Riemannian interclass discriminability and intraclass consistency	Track user skill	✓			Classifier-independent
	Duan et al. (2021)	Riemannian metrics (Lotte and Jeunet, 2018) + diffusion maps	Feedback	✓	✓	✓	Performance represented only visually; user infers performance changes from visualization

CA, classifier accuracy; M1, primary motor cortex; SMR, sensory motor rhythm.

A few metrics have been developed on the basis of short EEG recordings of the resting brain to prognosticate BCI inefficiency. These studies have shown that BCI classification accuracy is positively correlated with an sensorimotor rhythm (SMR) predictor, i.e., the maximum difference between the power spectral density curve during a relax with eyes open condition and a fit of the 1/*f* noise spectrum (Blankertz et al., 2010; Sannelli et al., 2019), a ratio of frequency band signal powers, i.e., (*P*_α_+*P*_β_)/(*P*_θ_+*P*_γ_) (Ahn et al., 2013b), and a single channel spectral entropy estimate over the motor cortex during rest (Zhang et al., 2015). Despite the potential of forecasting the accuracy of SMR BCI control, these metrics were not designed to provide user-feedback and do not satisfy the feedback metric design objectives.

A simple metric derived from EEG corresponding to active brain states is the classifier output (i.e., predicted mental task label) as initially developed by the Graz BCI group (Pfurtscheller et al., 2003). Since the initial use of classifiers for training, others have investigated alternative methods of using classifier feedback for training such as providing biased feedback (Barbero and Grosse-Wentrup, 2010; Alimardani et al., 2014) or breaking trials into multiple segments to be classified individually and providing positive feedback only if the individual windows have non-zero sensitivity during a trial (at least one true positive) and maximum specificity (zero false positives) in the periods immediately preceding or following a trial (Sburlea et al., 2015). Unfortunately, changes in classifier output may not reflect changes in user-performance (Lotte and Jeunet, 2018), can be difficult to interpret due in part to a lack of explanatory feedback (Jeunet et al., 2016a) or a lack of user understanding of algorithmic mechanics (Lotte et al., 2013; Müller et al., 2017), and conflates the performance of the classifier with that of the human user (Lotte and Jeunet, 2018). Other BCI user performance metrics derived from EEG during the execution of mental tasks do not directly relate to task performance as in Bamdadian et al. (2014), or require *a priori* determination of user-specific frequency bands, which may preclude real-time deployment during initial user training (Shu et al., 2018). Lotte and Jeunet (2018) proposed Riemannian geometry-based metrics (see Congedo et al., 2017; Yger et al., 2017 for reviews of Riemannian geometry for BCIs) to track changes in user motor imagery skill. Advantageously, these Riemannian metrics are agnostic to the selection of electrode channels and BCI classifier, and do not rely on user-specific hyperparameters. Nonetheless, these metrics were designed to characterize user performance on the basis of a static rather than a dynamic data set. As such, these metrics neither integrate new data nor reflect changes in user performance as new trials are attempted. Duan et al. (2021) rendered these metrics in conjunction with diffusion maps to provide a visual representation of the relative similarities and differences of recent trials to users during online training.

In this paper, we propose alterations to the Riemannian geometry-based metrics due to Lotte and Jeunet (2018) to admit new data during online training. These metrics were selected for further study due to their intuitive connection to trial performance and their freedom from user-specific hyperparameters.

## 2. Materials and methods

### 2.1. Performance metric design

We formulate dynamic variations of the “class distinctiveness” (*classDistinct*) and “class stability” (*classStability*) metrics introduced by Lotte and Jeunet (2018). We first define inter- and intra-class dispersion, as they are fundamental to the computation of the metrics. The inter-class dispersion was defined as the distance between class mean covariance matrices:


(1) $$interClassDisp(\bar{\Gamma })=\delta_{R}({\bar{\Gamma }}_{1},{\bar{\Gamma }}_{2})$$

where ${\bar{\Gamma }}_{c}$ is the mean covariance matrix for class *c* and δ_*R*_ denotes the Riemannian distance. Note that Riemannian mean covariance matrix is estimated using numerical methods as no closed form solutions are known (Congedo et al., 2017; Yger et al., 2017). Intra-class dispersion, Φ_*c*_, was computed using the mean distance between covariance matrices of individual trials from class *c* and the mean covariance matrix of class *c*:


(2) $$\Phi_{c}=\frac{1}{N_{c}}\sum_{i=1}^{N_{c}}\delta_{R}({\bar{\Gamma }}_{c},\Gamma_{c,i})$$

where ${\bar{\Gamma }}_{c}$ is the mean covariance matrix for class *c*, Γ_*c, i*_ is the *i*th trial covariance matrix from class *c*, *N*_*c*_ is the number of trials belonging to class *c*, and δ_*R*_ is again the Riemannian distance.

The *classDistinct* metric was defined as the ratio of inter- to intra-class dispersions:


(3) $$classDistinct(\Gamma ,\Phi )=\frac{\delta_{R}({\bar{\Gamma }}_{1},{\bar{\Gamma }}_{2})}{\Phi_{1}+\Phi_{2}}.$$

while the *classStability* of class *c* was computed as the inverse of the cognate intra-class dispersion, namely,


(4) $$\begin{array}{l} classStability\left(\Phi_{c}\right)=\frac{1}{1+\Phi_{c}}. \end{array}$$

Note that these equations apply to two-class BCIs; however, they can be extended to more than two classes (Lotte and Jeunet, 2018). As defined here, metrics (3) and (4) use a static set of trials to assess user performance at a particular point in time. To ensure that incremental, time-dependent feedback is available shortly after each new trial and to provide an indication of whether performance is improving over time, we propose three different methods of dynamically updating these metrics.

Consider that there are *N*_*C*_ classes or separate BCI tasks and that a session contains *N* blocks (or runs). Let ${\bar{\Gamma }}_{k,c}$ refer to the sessional mean covariance matrix for BCI class *c*, *c* = 1, …, *N*_*C*_ after block *k*, *k* = 1, …, *N*. Φ_*k, c*_ refers to the sessional intra-class dispersion for class *c* after block *k* and ϕ_*k, c*_ refers to mean deviation from the mean for the class *c* trials of block *k*.

The first trial-wise adaption method (hereafter referred to as the **running** *classDistinct*/*classStability* method) adds each new trial to a set of trials from the previous *k*−1 blocks and current *k*th block and recomputes (3)–(4). While simple to implement, this method has potential disadvantages. Primarily, as there will generally be a greater proportion of trials from previous blocks, the changes in the metrics due to new trials may be muted. Furthermore, with respect to the *classStability* metric, this method may penalize users for exploring different mental task strategies by artificially inflating the intra-class dispersion.

The second proposed approach is to use a **sliding window** of trials to compute the metrics. With this approach, a fixed-length queue of the most recent trials for each class are retained, ensuring that past trials do not unduly influence metric values. After a new trial is added to (and the oldest trial removed from) the queue, (1)–(4) are recomputed using the trials within the queue.

**Figure 1:**
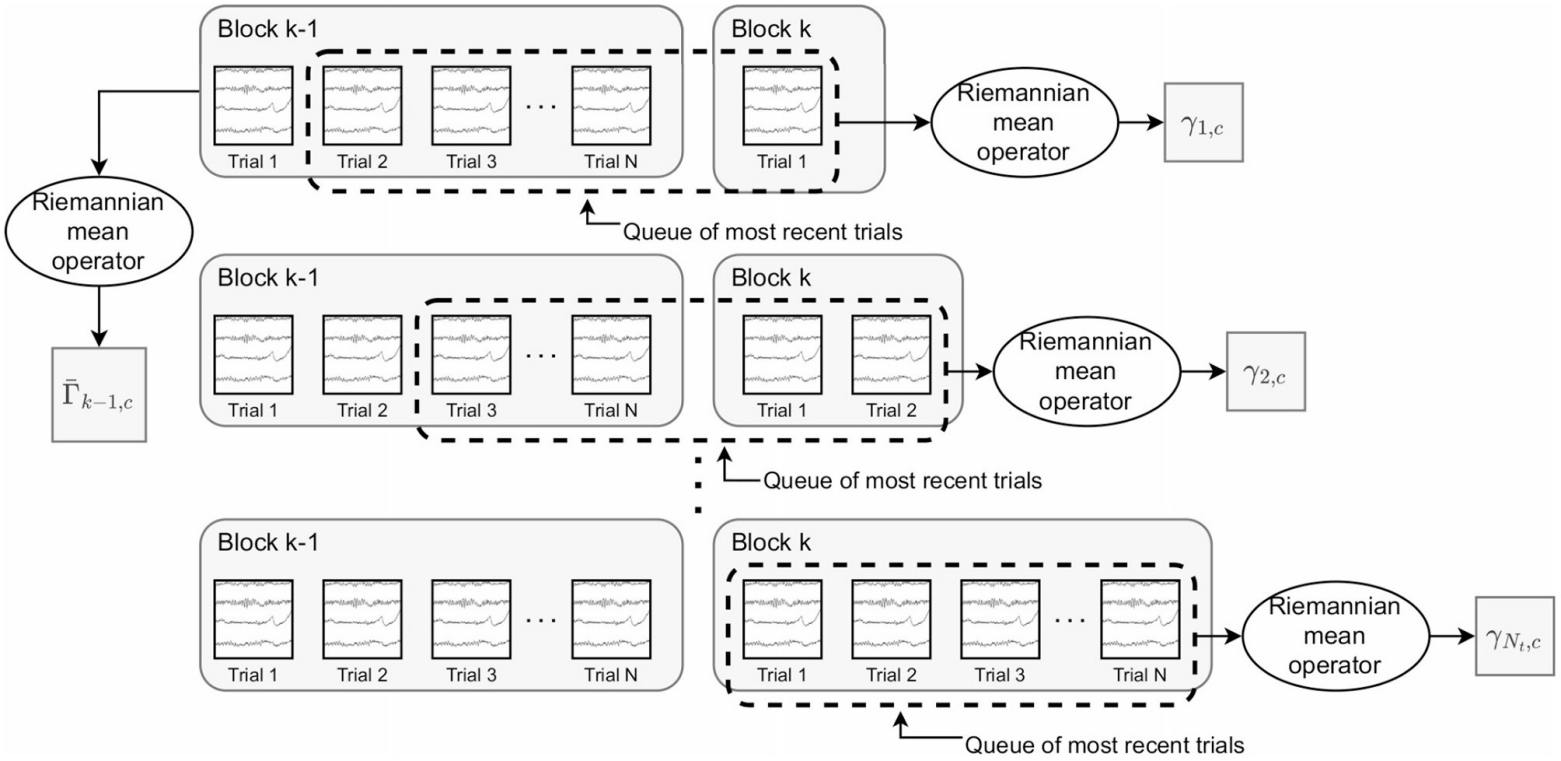
Depiction of the trials used to compute ${\bar{\Gamma }}_{k-1,c}$ (the mean covariance matrix for class *c* based on trials from the previous block) and γ_*i, c*_ (the mean covariance matrix for the most recent trials for class *c* after the *i*th trial). These are the key quantities in the weighted average *classDistinct* metric. ${\bar{\Gamma }}_{k-1,c}$ remains constant as the trials of the *k*th block are performed. The trials within the dashed rectangle represent the queue of the most recently performed trials. γ_*i, c*_ is updated using the trials within the queue after each new trial within the *k*th block.

The final proposed approach is to use a **weighted average** of past and recent trials for both the post-trial mean and intra-class dispersion. Queues are again utilized to maintain a set of the most recent trials for each class. However, the post-trial mean is computed as:


(5) $${\bar{\Gamma }}_{k-1,c}^{\frac{1}{2}}{\left({\bar{\Gamma }}_{k-1,c}^{-\frac{1}{2}}\gamma_{i,c}{\bar{\Gamma }}_{k-1,c}^{-\frac{1}{2}}\right)}^{\alpha_{1}}{\bar{\Gamma }}_{k-1,c}^{\frac{1}{2}}$$

where ${\bar{\Gamma }}_{k-1,c}$ is the mean for class *c* from the previous block of trials and γ_*i, c*_ is the mean of the most recent trials for class *c* within the queue upon completion of the *i*th trial within the *k*th block (Figure 1). α_1_∈[0, 1] is a constant controlling the relative weights of the two means. This update equation is equivalent to the convex sum $\left(1-\alpha_{1}\right){\bar{\Gamma }}_{k-1,c}+\alpha_{1}\gamma_{c}$ in Euclidean geometry (Congedo et al., 2017). At the beginning of a block, the queue contains primarily trials from the previous block and thus, the sets of trials used to compute Γ_*k*−1, *c*_ and γ_*i, c*_ have large intersection. As more trials are completed, the relative influence of trials from the previous block are more gradually reduced than in the moving average approach. For the weighted average *classDistinct* and *classStability* metrics, we made the following modification to the calculation of the intra-class dispersion. We split the set of trials, *T*, into *N*_*s*_ subsets of *N*_*t*_ trials, *T*_*j*_, such that


$$T_{1}\cup T_{2}\cup \cdots \cup T_{N_{s}}=T.$$

Subsets were formed by splitting trials according to the chronological order in which they were performed; for example, the first *N*_*t*_ trials performed during a block would be grouped into subset *T*_1_. Using these subsets, we computed a modified intra-class dispersion as:


$$\begin{array}{l} \Phi^{*}=\frac{1}{N_{s}}\frac{1}{N_{t}}\overset{N_{s}}{\underset{j=1}{\sum }}\overset{N_{t}}{\underset{i=1}{\sum }}\delta_{R}\left({\bar{\Gamma }}_{T_{j}},\;\Gamma_{T_{j,i}\;}\right)\; \end{array}$$

where *N*_*s*_ is the number of trial subsets, *N*_*t*_ is the number of trials in each subset, ${\bar{\Gamma }}_{T_{j}}$ is the mean covariance matrix of trials within the *j*^*th*^ subset of trials, Γ_*T*_*j, i*__ is the covariance matrix of the *i*^*th*^ trial within subset *T*_*j*_, and δ_*R*_ denotes the Riemannian distance. The motivation behind this modification was to reduce the impact of signal non-stationarities that may artificially increase the intra-class dispersion when considering a large number of trials. For our analysis, we set *N*_*t*_ = 5. Trial subsets were disjoint save for when computing within-block post-trial intra-class dispersion values. If the number of trials completed within the block was not divisible by *N*_*t*_, subset *T*_*N*_*s*__ was formed using the most recently completed *N*_*t*_ trials; consequently, this subset could share up to *N*_*t*_−1 trials with subset *T*_*N*_*s*_ − 1_.

The post-trial intra-class dispersion was computed using this modified intra-class dispersion:


(6) $$\begin{array}{c} \left(1-\alpha_{2}\right)\Phi_{k-1,c}^{*}+\alpha_{2}\phi_{k,c}^{*} \end{array}$$

where α_2_∈[0, 1] is a constant, ϕ_*k*−1, *c*_ is the intra-class dispersion for the class *c* trials of the (*k*−1)th block, and ϕ_*k, c*_ is the intra-class dispersion of class *c* trials completed only during the current (*k*th) block. This estimate does not artificially penalize users for exploring different mental task strategies among blocks as means and mean deviations from the means are computed for trials within each block independently before being combined.

### 2.2. Experimental design

The experimental goal was to assess how accurately trial-wise reinforcement signals reflect longer-term (i.e., over the course of several trials) performance changes. The term “reinforcement signals” will be used rather than “feedback” because our focus in this study was to assess the numerical values that would be used to generate feedback; we did not evaluate how to present the reinforcement signals to users nor how these would be interpreted by users.

To quantify “longer-term performance changes,” we computed performance metrics for non-overlapping blocks of 40 trials (20 per class) and then computed a “block-wise performance change” by computing the change in different metrics between adjacent blocks. Note that computing performance metrics in this manner with disjoint sets of trials is often how user performance is tracked in BCI studies (e.g., Vidaurre et al., 2011a; Lotte and Jeunet, 2018; Meng and He, 2019).

**Figure 2:**
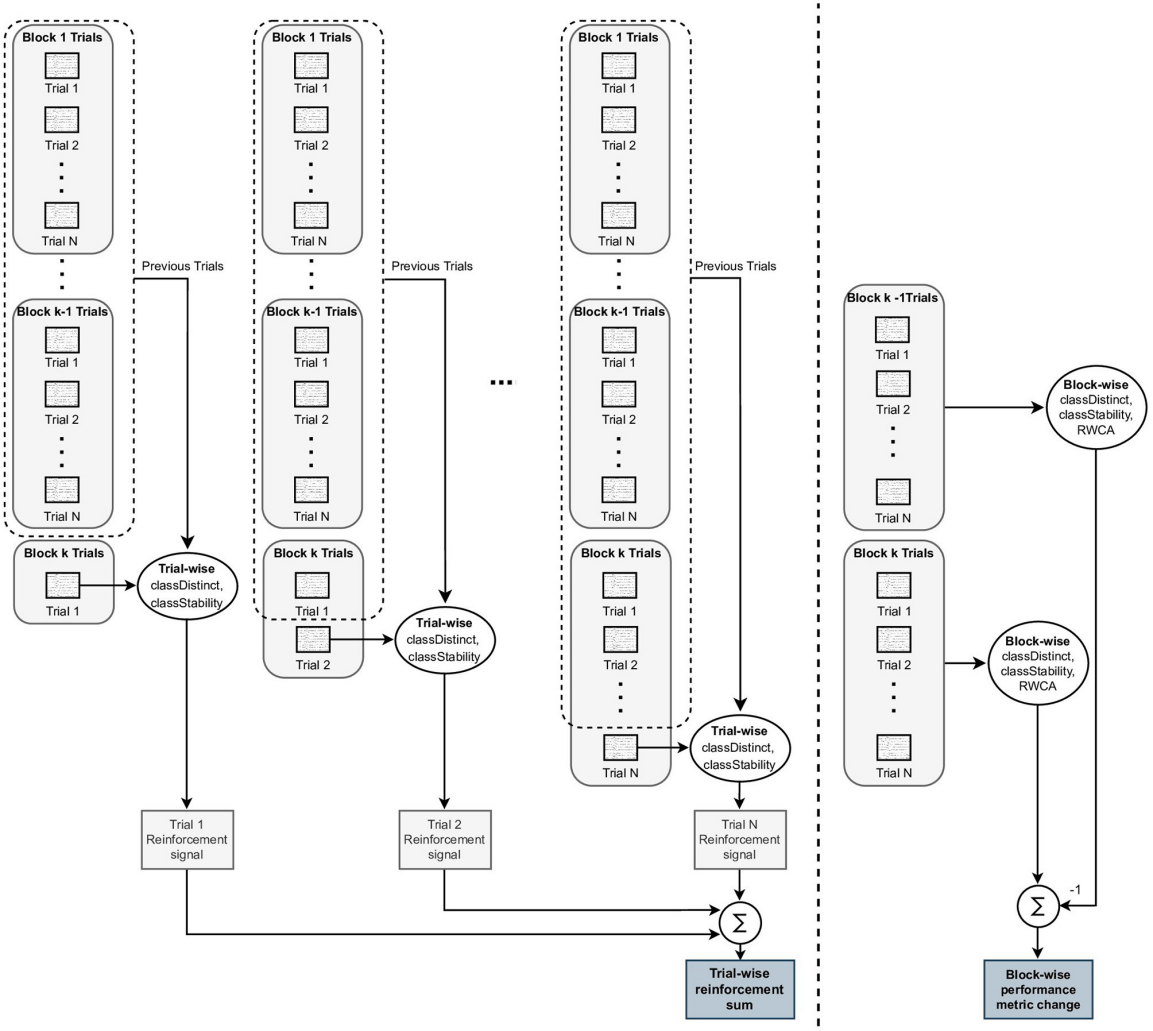
Illustration of block-wise performance change and trial-wise reinforcement signal sum calculations. To the left of the dashed line, the single trial reinforcement signals are computed using the *i*th trial within a block and the set of all previously performed trials. The trial-wise reinforcement signals for each trial within the block are summed to generate a trial-wise reinforcement sum for the block. To the right of the dashed line, the Block-wise metrics, *classDistinct, classStability*, and run-wise classification accuracy (RWCA, see Section 2.5) are computed using all of the trials within a single block, and the block-wise performance change is computed as the difference between the *k*th and (*k*−1)th block-wise metric values. The objective of this study was to evaluate how closely the trial-wise reinforcement sums reflected the block-wise performance changes.

To assess the level of agreement between the trial-wise reinforcement signals and the block-wise changes, we computed a reinforcement signal for each trial within a block, then summed all these individual trial reinforcement signals, and finally compared the sum of trial-wise reinforcement signals to the block-wise change. More specifically, we evaluated the extent to which: (i) trial-wise reinforcement signal sums correlated to performance changes computed over a block of trials and (ii) trial-wise reinforcement signal sums correctly discriminated between positive and negative block-wise performance changes. The calculation of block-wise performance changes and trial-wise reinforcement signal sums is summarized in Figure 2.

Sums of the trial-wise reinforcement signals were used in the analysis rather than the single trial reinforcement signals as the intent was to investigate the cumulative reinforcement over a larger set of trials. In practice, the cumulative reinforcement is likely to be a more reliable training tool than single-trial feedback, which can be sensitive to noise and other spurious factors (e.g., covariate shifts).

### 2.3. Simulated EEG data

Simulated EEG data were generated using the simBCI software library (Lindgren et al., 2018). Simulation parameters were set according to BCI Competition IV data generation example described in Lindgren et al. (2018) with the following modifications: trial lengths were set to 4,000 ms, sampling frequency set to 250 Hz, and all eye movement/blink effects removed. All simulations were run using the MRI volume-derived leadfield model contained in the “leadfield-mediumRefinement.mat” file available for download with the simBCI software. All analyses were performed on electrodes 18, 33, 47, 84, 91, 104, 145, 188, and 218 approximating positions Fz, F4, C4, Cz, P4, Pz, P3, C3, and F3, respectively.

BCI session blocks were generated to simulate three levels of BCI-user performance: (i) low, (ii) moderate, and (iii) high performance. Table 2 outlines the characteristics of each of these performance levels. 100 blocks of 40 trials (20 per class) for each of these performance levels were generated. Pairs of these blocks were then used to form 700 simulated two-block BCI sessions. To simulate changing performance conditions, 100 sessions of each of the following block pairs were created: low-moderate, low-high, moderate-low, moderate-high, high-low, and high-moderate (hereafter abbreviated as LM, LH, ML, MH, and HM respectively). Another 100 sessions were created using low-low, moderate-moderate, high-high pairs (abbreviated as NC, for “no change”) to simulate constant performance conditions. Table 3 provides a summary of each of the simulated session types.

**Table 2:** Descriptions of the three simulated performance levels.

**Performance level**	**Description**	**Interpretation**
Low	Both “left” and “right” hand motor imagery tasks result in ERDs in the right motor cortex	A user who produces similar EEG patterns for multiple tasks
Moderate	“Left” motor imagery tasks consistently produce ERDs in the right motor cortex while “right” motor imagery tasks elicit a left motor cortex ERD in half the trials and a right motor cortex ERD in the other half	A user who has achieved good performance with one task but lacks consistency with a second task
High	“Left” and “right” motor imagery tasks consistently trigger a contralateral ERD	A user with idealized performance

ERD, event-related desyncrhonization.

**Table 3:** Simulated session types.

**Name**	**Block 1 performance level**	**Block 2 performance level**
LM	Low	Moderate
LH	Low	High
ML	Moderate	Low
MH	Moderate	High
HL	High	Low
HM	High	Moderate
NC	Low, moderate, or high	Same as block 1

These simulated sessions were not intended to represent realistic BCI-user learning capabilities; indeed, such pronounced performance changes would be unlikely in adjacent blocks with real users. Rather these simulated sessions provide meaningful and controlled scenarios which can be used to evaluate the response of the different metrics to performance changes.

Prior to analysis, the EEG data were zero-phase filtered using fourth order Butterworth filters with a passband of 8–30 Hz. Data were epoched into trial segments consisting of the middle two seconds of the four second trial.

### 2.4. Real EEG data

The proposed metrics were also evaluated using previously published (Cho et al., 2017) SMR-EEG data (52 subjects, left and right hand motor imagery tasks, 64 Ag/AgCl active electrodes). Each participant completed 100 or 120 trials of each motor imagery task in blocks of 40 trials (20 per task). Within each seven second trial, participants were instructed to perform a three second imagined left or right hand finger movement sequence. At the end of each block of trials, participants were given the classification results of the latest block of trials. Full participant and instrumentation details are provided by Cho et al. (2017).

We considered data from a subset of channels, namely, F3, Fz, F4, C3, Cz, C4, P3, Pz, and P4. All data were zero-phase filtered using fourth order Butterworth filters with a passband of 8–30 Hz. Subsequently, all data were downsampled from 512 to 256 Hz. All analysis was performed using the central two seconds of the three second motor imagery tasks.

Finally, as our Riemannian geometry-based performance metrics are sensitive to artifacts, a two stage procedure was applied to remove trials containing artifacts. The first step was to remove any of the trials flagged as containing measurement or movement artifacts by Cho et al. (2017). In the second step, the offline Riemannian Potato Field signal quality procedure (Barthélemy et al., 2019) was applied. Seven individual “potatoes” were defined according to the recommendations from Barthélemy et al. (2019): five electrode contact loss detectors using paired electrode channels (F3-C3, P3-Pz, Fz-F4, Cz-C4, and C4-P4) bandpass filtered between 1 and 20 Hz and three general artifact detectors using groups of four electrodes (F3-C3-P3-Pz, Fz-F4-Cz-C4, and P4-Pz-Fz-F4) bandstop filtered between 8 and 38 Hz. Each potato filter was calibrated using a subject's entire set of trials. Any trial identified by the algorithm as containing artifacts was excluded from further analysis.

### 2.5. Block-wise performance evaluation

The *classDistinct* and *classStability* metrics were computed for blocks of 40 trials (20 per class) for each simulated session and for each individual in the real dataset. An overall *classStability* metric was constructed by calculating the average of the metric between imagery tasks.

An additional block-wise performance metric, inspired by the run-wise classification accuracy (RWCA) metric (Lotte and Jeunet, 2018), was computed. Using only trials from each individual block, we computed leave-one-trial-out cross-validation classification accuracy with a common spatial pattern feature-regularized linear discriminant analysis classifier (CSP-rLDA) (Blankertz et al., 2008). The CSP feature extraction pipeline comprised four spatial filters.

### 2.6. Trial-wise reinforcement calculations

Trial-wise reinforcement signals were derived from classifier output and our proposed running, sliding window, and weighted average *classDistinct* and *classStability* metrics for blocks of simulated and real EEG. The sum of the trial-wise reinforcement signals for each method was then computed for each block of trials. For the weighted average *classDistinct* (5) and *classStability* (6) metrics, α_1_ and α_2_, were both set to 0.9.

Trial-wise reinforcement signals for the *classDistinct* and *classStability* metrics were computed as:


$$M_{k,i}^{\prime }-M_{k-1}$$

where $M_{k,i}^{\prime }$ is the value of the metric after incorporating the *i*th trial within the *k*th block according to the procedures outlined in Section 2.1, and *M*_*k*−1_ is the corresponding value of *classDistinct* or *classStability* computed using the trials from the (*k*−1)th block. The accumulation of reinforcement signals for each block yielded the trial-wise reinforcement sum for the block. Under this reinforcement calculation scheme, users would be given positive or negative feedback when the metrics were increasing or decreasing, respectively, relative to the start of the block. The metric value at the start of the block, rather than the value after the previous trial, was used as the reference point to emphasize gradual trends in performance rather than potentially volatile trial-wise changes.

Single trial classifier reinforcement outputs were counted as either +1 (reward for correct prediction) or −1 (punishment for incorrect prediction). Classifier outputs for the *k*th block in a session were generated using CSP-rLDA classifiers trained using data from the participant's previous *k*−1 blocks. All CSP feature extraction used four spatial filters. A three-fold cross-validation on the training data identified the best temporal sub-band (8–11 Hz, 9–13 Hz, 11–19 Hz, 17–30 Hz or 8–30 Hz) for classification.

### 2.7. Statistical analysis

For simulated EEG data, the agreement between block-wise metric changes and the different trial-wise reinforcement sums was estimated by Spearman correlation whereas for real EEG data, repeated measures correlations (Bakdash and Marusich, 2017), which controls for inter-participant variance, was invoked. Using 1,000 bootstrap samples, we generated 95% confidence intervals for the difference between coefficients, e.g., *r*_*weighted*−*avg*_−*r*_*running*_. These confidence intervals were Bonferroni-corrected to maintain a family-wise type I error of 5%. Significant differences were identified if the confidence intervals did not include 0.

To evaluate the extent to which the reinforcement sums could be used to discriminate between positive or negative block-wise changes in performance, we generated empirical receiver-operator characteristic (ROC) curves, with the sign of the block-wise metric change serving as the ground truth label and the trial-wise sums as the discriminating signal. The area under the ROC curve (AUC) for the different metrics were compared using DeLong's test (DeLong et al., 1988; Robin et al., 2011).

The ROC curves indicate the discrimination ability of the reinforcement sums with arbitrary boundary thresholds. However, in practice a threshold of zero would likely be more intuitive for users to interpret. Therefore, we also compared the proportion of reinforcement sums which had the same sign as the block-wise metric change using McNemar's exact tests (McNemar, 1947; Agresti, 2003). Unless stated otherwise, the Holm method (Holm, 1979) was applied to adjust all *p* values for multiple pairwise comparisons.

## 3. Results

### 3.1. Correlation between trial-wise reinforcement signal sums and block-wise performance change

**Figure 3:**
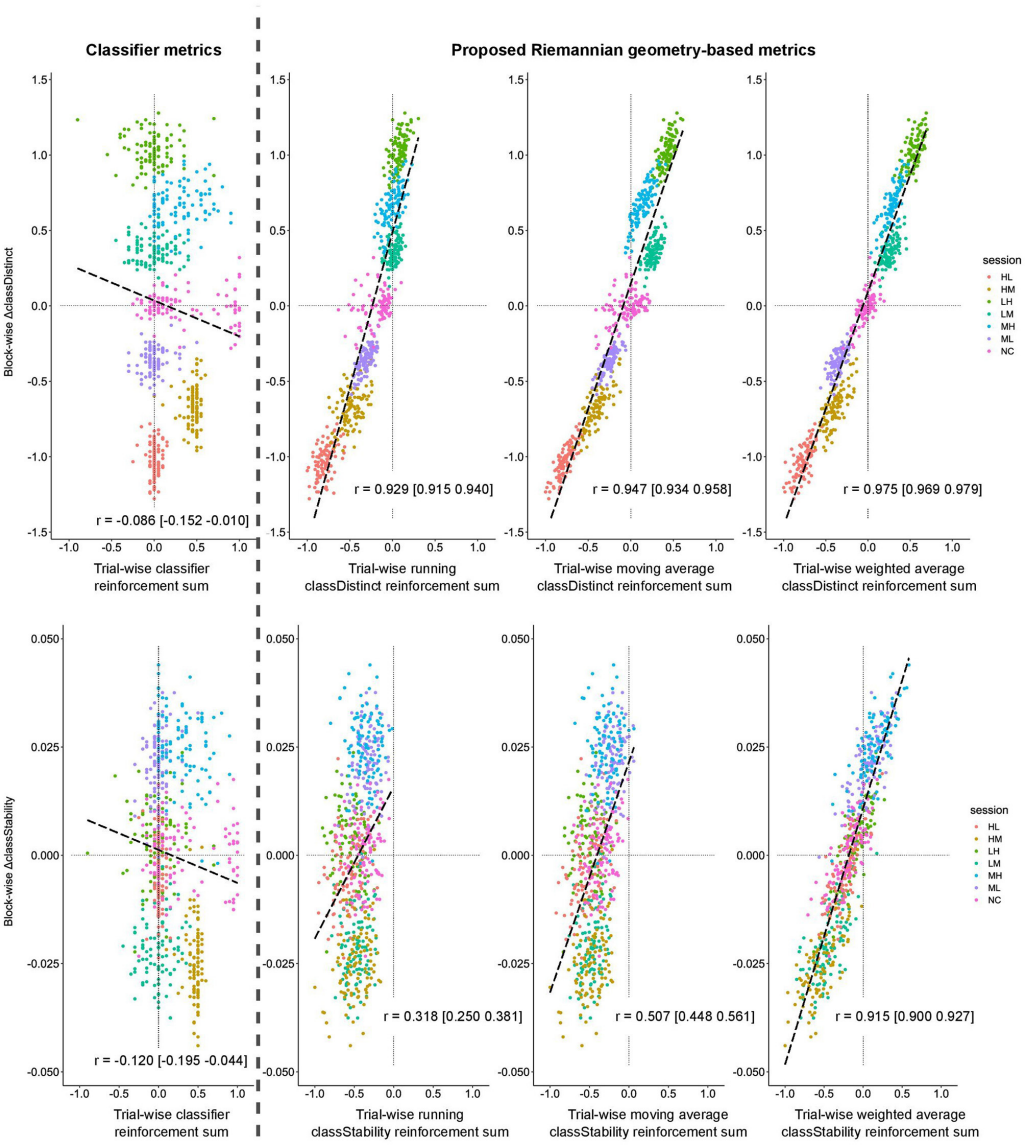
Block-wise change in *classDistinct* **(top row)** and *classStability* **(bottom row)** for different trial-wise reinforcement signals from simulated data sessions. All trial-wise reinforcement sums are normalized to [−1, 1]. Long dashed lines represent lines of best fit. Vertical gray dashed line separates classifier reinforcement sums **(left)** and *classDistinct*/*classStability* reinforcement sums **(right)**.

Figure 3 plots the block-wise change in the *classDistinct* (top row) and *classStability* (bottom row) metrics against their cognate sum of trial-wise reinforcement signals for the simulated EEG data. In the *classDistinct* case only, discernible clusters for each session type emerged according to the simulated changes in user performance (i.e., lower reinforcement values for decreasing performance as in ML, HM, and HL sessions, and higher reinforcement values for increasing performance as in LM, MH, and LH sessions). In contrast, for both metrics, the reinforcement sums for the classifier output generally hovered around zero regardless of simulated user performance.

Block-wise changes for both metrics were positively correlated (*p* < 0.05) with their cognate running, sliding window, and weighted average trial-wise reinforcement sums. In contrast, the block-wise changes were slightly negatively correlated (*p* < 0.05) with trial-wise classifier output reinforcement sums. All pairwise comparisons between reinforcement sums were significant (*p* < 0.05). Similar relationships were observed for block-wise RWCA changes.

**Figure 4:**
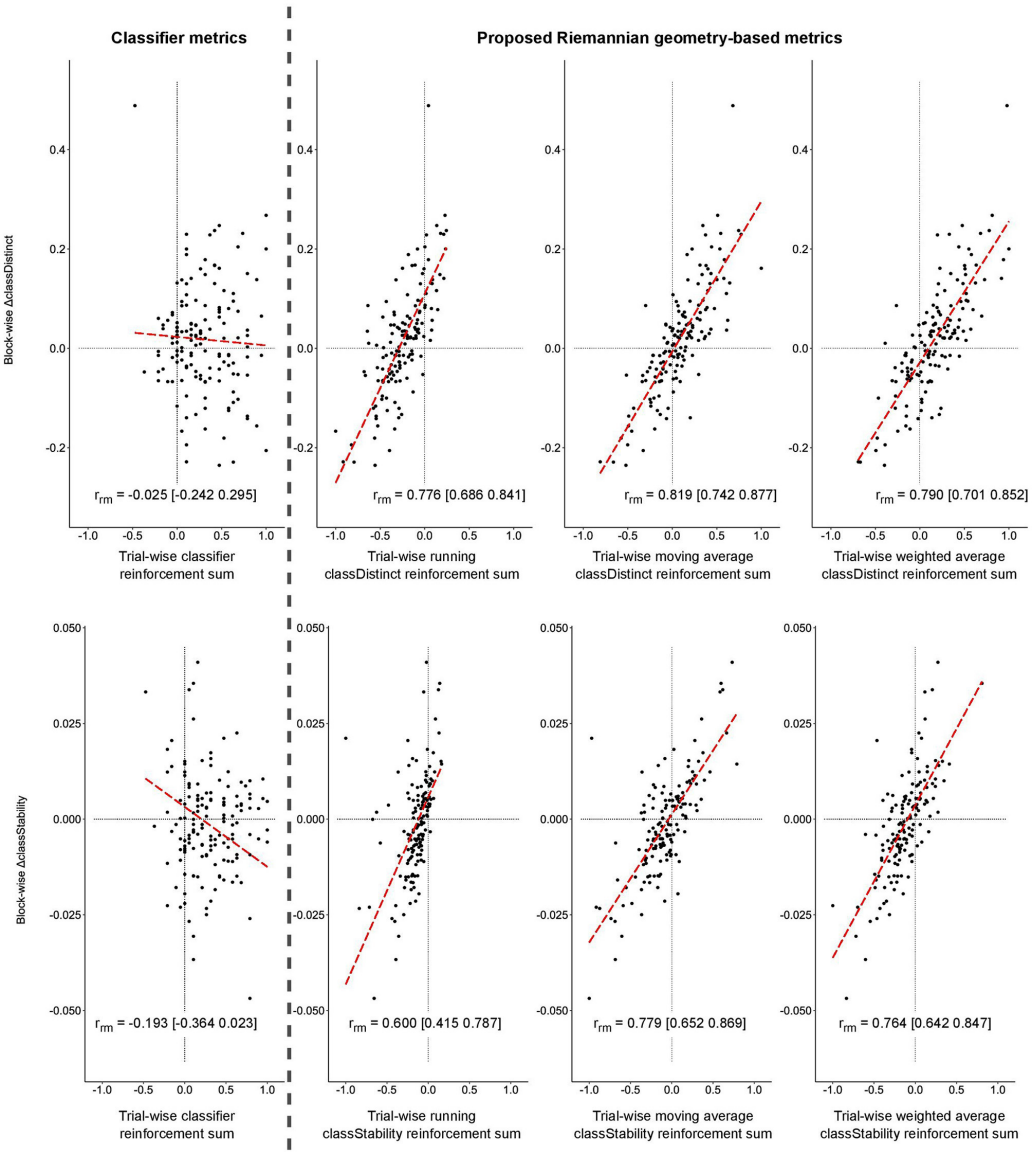
Block-wise change in *classDistinct* **(top row)** and *classStability* **(bottom row)** for different trial-wise reinforcement signals from real EEG data. All trial-wise reinforcement sums are normalized to [−1, 1]. Long dashed lines represent lines of best fit. Vertical gray dashed line separates classifier reinforcement sums **(left)** and *classDistinct*/*classStability* reinforcement sums **(right)**.

Similar significant relationships between the block-wise changes in *classDistinct* and *classStability* and trial-wise reinforcement sums were observed for the real EEG data, as depicted in Figure 4. Correlations involving trial-wise *classDistinct* and *classStability* reinforcement sums remained significantly greater (*p* < 0.05) than those for classifier reinforcement.

### 3.2. Trial-wise reinforcement sum discriminatory ability

**Figure 5:**
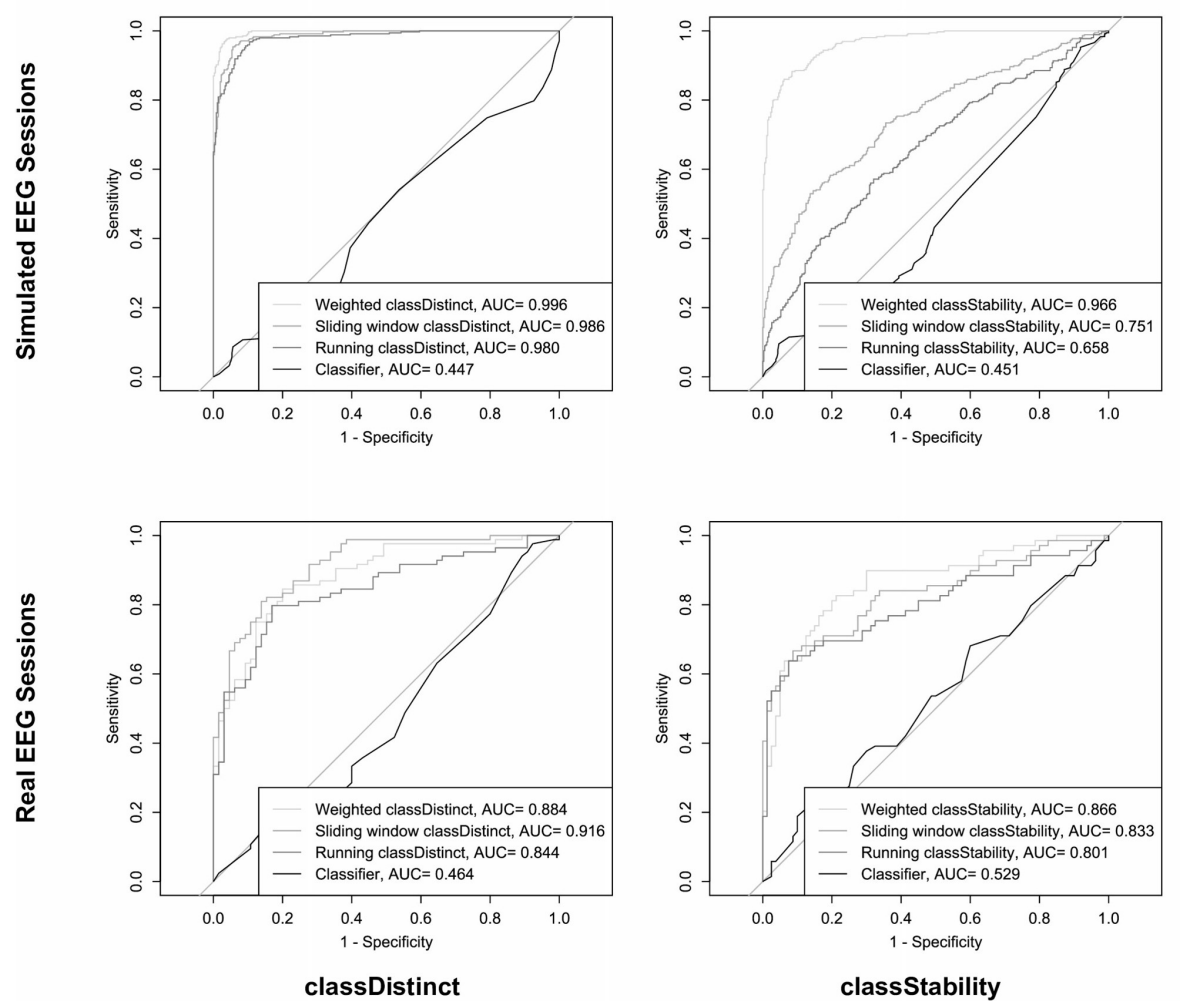
ROC curves and AUC values for prediction of the sign of block-wise *classDistinct* **(left column)** and *classStability* **(right column)** changes, using different trial-wise reinforcement sums in simulated **(top row)** and real **(bottom row)** EEG sessions.

The top row of Figure 5 shows the ROC curves and corresponding AUC values for discriminating the sign of the block-wise changes in *classDistinct* (left) and *classStability* (right) using different trial-wise reinforcement sums for the simulated EEG sessions. The AUC values in the upper left graph of Figure 5 indicate that *classDistinct* reinforcement sum variants could discriminate positive from negative block-wise changes with arbitrary (i.e., non-zero) thresholds. AUC values in the upper right graph of Figure 5 reveal that weighted *classStability* reinforcement outperformed the other metrics for discriminating the sign of block-wise *classStability* changes. Pairwise differences between AUC values were significant for both *classDistinct* (*p* < 0.05, Delong's tests) and *classStability* (*p* < 0.0001, Delong's tests) ROC curves.

The bottom row of Figure 5 shows the ROC and AUC curves for discriminating the sign of the block-wise changes in *classDistinct* (left) and *classStability* (right) using the different trial-wise reinforcement sums in the real EEG-BCI sessions. AUC values for predictions of block-wise metric changes using trial-wise classifier reinforcement were significantly smaller than those of the other reinforcement metrics (*p* < 0.0001) in both *classDistinct* and *classStability* cases. Sliding window *classDistinct* AUC values were significantly larger than the running *classDistinct* metrics (*p* < 0.01).

The ability to discriminate block-wise performance changes using a fixed threshold of zero varied across the proposed Riemannian geometry metrics. Table 4 indicates in bold the number of times that the signs of trial-wise reinforcement sums agreed with the signs of block-wise changes for different metrics.

**Table 4:** Cross-tabulation of the sign of block-wise changes and the sign of the trial-wise reinforcement sums, for simulated and real EEG data sets, and for *classDistinct* and *classStability* metrics.

**Data**	**Metric**	**Block-**	**Reinforcement**							
		**wise**	**Classifier**		**Running**		**Sliding window**		**Weighted average**	
		**change**	**Pos**.	**Neg**.	**Pos**.	**Neg**.	**Pos**.	**Neg**.	**Pos**.	**Neg**.
Simulated	*classDistinct*	Pos.	**187**	87	**195**	151	**318**	28	**335**	11
		Neg.	190	**74**	0	**354**	18	**336**	11	**343**
	*classStability*	Pos.	**183**	89	**0**	357	**6**	351	**198**	159
		Neg.	194	**72**	0	**343**	0	**343**	1	**342**
Real	*classDistinct*	Pos.	**65**	9	**24**	60	**76**	8	**78**	6
		Neg.	52	**9**	0	**65**	18	**47**	29	**36**
	*classStability*	Pos.	**55**	8	**21**	48	**46**	23	**41**	28
		Neg.	62	**10**	1	**79**	8	**72**	4	**76**

Bold numbers indicate sign alignment between the block-wise metric changes and a trial-wise reinforcement sums. Sums precisely equal to zero were omitted from the counts.

For simulated EEG data, McNemar's tests revealed that all pairwise comparisons of counts between different reinforcement sums were significant (*p* < 0.0001). The running *classDistinct* reinforcement sum had a bias toward smaller/more negative sums, resulting in lower agreement with the sign of the corresponding block-wise change. The differences between the sliding window and weighted average proportions all emerged from the MH and NC sessions. The weighted average *classStability* trial-wise reinforcement sum appeared to be the most effective in discriminating the sign of the block-wise *classStability* change (Table 4; Figure 3). Exact McNemar's tests revealed that the counts for the weighted average *classStability* reinforcement sums were significantly different from the counts for each of the other three reinforcement sums (*p* < 0.0001). Both the running and sliding window *classStability* reinforcement sums appeared to have a strong bias toward negative values, with the sum being negative in 700 and 694 of the blocks for the running and sliding window variants, respectively.

In the real EEG data, the sliding window and weighted average *classDistinct* and *classStability* trial-wise sums outperformed the corresponding classifier and running reinforcements (Table 4; *p* < 0.05, exact McNemar's tests).

## 4. Discussion

### 4.1. Sensitivity to performance changes: Trial-wise Riemannian metrics outperform classifier output

The correlation and ROC curve analyses demonstrated that classifier-based reinforcement failed to reflect the block-wise trend in user performance changes. This result in itself is unsurprising as the CSP-rLDA classifier, like other commonly deployed SMR-BCI classifiers, relied on the assumption of stationary and consistent data distributions. In the presence of time-dependent distributions, the classifier is ill-equipped to track changes in class distributions. The proposed Riemannian metrics, on the other hand, dynamically update inter- and intra-class dispersion estimates and as a result are more suited to detecting changes to distributions. The *classDistinct* and *classStability* metrics yield continuous-valued reinforcement signals. This means that small distributional changes due to noise are muted in comparison to larger data shifts due to evolving ERD activations, thereby, assigning more weight to changes that are most relevant to the user. In contrast, with predicted class label feedback, each trial is equally weighted as a correct or incorrect prediction.

Our results concerning classifier feedback, however, should not be interpreted as being irreconcilable with findings of others who have observed that classifier-based feedback can be harnessed to improve performance (e.g., Vidaurre and Blankertz, 2010; Müller et al., 2017; Meng and He, 2019). In cases where users are at least moderately proficient and data distributions are stable, users could plausibly observe changes in their block-wise classification accuracies that guide them toward improved performance. However, our results accentuate the conclusions of others (Lotte et al., 2013; Jeunet et al., 2016a) that classifier feedback is particularly challenging to utilize for initially poor performers, in part, because their data are poorly separable and consequently, classifier output will likely appear random, even when separability of the data is improving. In light these findings, future research may investigate a hybrid feedback approach where the Riemannian metrics are deployed during the early stages of training to facilitate user exploration, until a moderate level of user performance is achieved, at which point traditional classifier feedback could be introduced to support the fine tuning of mental activity.

### 4.2. Accurately reinforcing block-wise performance trends: Sliding window and weighted Riemannian metrics yield favorable results

In comparison to the other proposed variants, the running *classDistinct* and *classStability* metrics had a bias toward lower, more negative reinforcement sums, resulting in lower discrimination of the direction of performance changes. The precise origin of this phenomenon is unclear. One potential hypothesis is that the estimated class mean covariance matrices may have overfit the noise within the set of individual trials. As the set of trials used to estimate the mean expands, the estimated mean becomes less sensitive to noise within individual trials. Consequently, the *classStability* would tend to decrease as overfitting subsides. Further, spurious differences between class means due to noise would decrease, causing inter-mean distances to converge and *classDistinct* to decrease. Because the running *classDistinct* and *classStability* reinforcement sums had higher probability of being negative, providing feedback based on these metrics could be detrimental. For lower-performing users, consistent negative reinforcement could be discouraging or frustrating (Lotte et al., 2013). Such changes to mental state and engagement with the technology could induce degradation in the performance of the BCI (Hammer et al., 2012; Ahn and Jun, 2015; Myrden and Chau, 2015).

With the simulated data, the sliding window *classStability* reinforcement sum also tended to negative values and poorly discriminated the sign of the block-wise metric change. This could in part be attributed to the composition of the simulated sessions. The HL and LH sessions had approximately constant consistency when analyzed at a block-wise level; however, in both of these sessions, a significant shift in one of the class means occurred between sessions. Within the block, therefore, the sliding window class covariance matrix moved slowly away from the previous block's cluster to the current block's cluster, resulting in inflated estimates of intra-class dispersion. The ML and MH sessions also exhibited this phenomenon, thereby masking stability improvements.

The sliding window *classStability* reinforcement sums also tended to be negative in the NC sessions where a shift in the mean was not due to variation in task performance or location of event-related desynchronization. Further investigation showed that the bias toward negative sums were driven by covariate shifts between blocks of the data generated by the simulator. As a result, the distributions of SMRs and noise had higher similarity within trials in the same block and thereby fueled the shifts in the means over time and inflated the sliding window *classStability*. The weighted *classStability* reinforcement metric was less sensitive to these effects because it computed individual covariance means for trials from their respective blocks and then calculated a weighted average of the variances about these means. In contrast, the two variants performed comparably with the real data suggesting that both may be suitable in scenarios where user performance changes more gradually between blocks and large inter-recording session covariate shifts are not present. Nonetheless, as such covariate shifts in data distributions are well documented in EEG-BCIs (e.g., Shenoy et al., 2006; Li et al., 2010; Raza et al., 2016), the finding of inflated *classStability* highlights a potential limitation of the sliding window *classStability* metric, particularly if the sliding window spans trials performed over multiple recording sessions. Alternatively, such distribution drift-induced metric changes could potentially be mitigated *via* adaptive rebiasing (Benaroch et al., 2021) to reduce inter-block and inter-session covariance distribution shifts.

**Figure 6:**
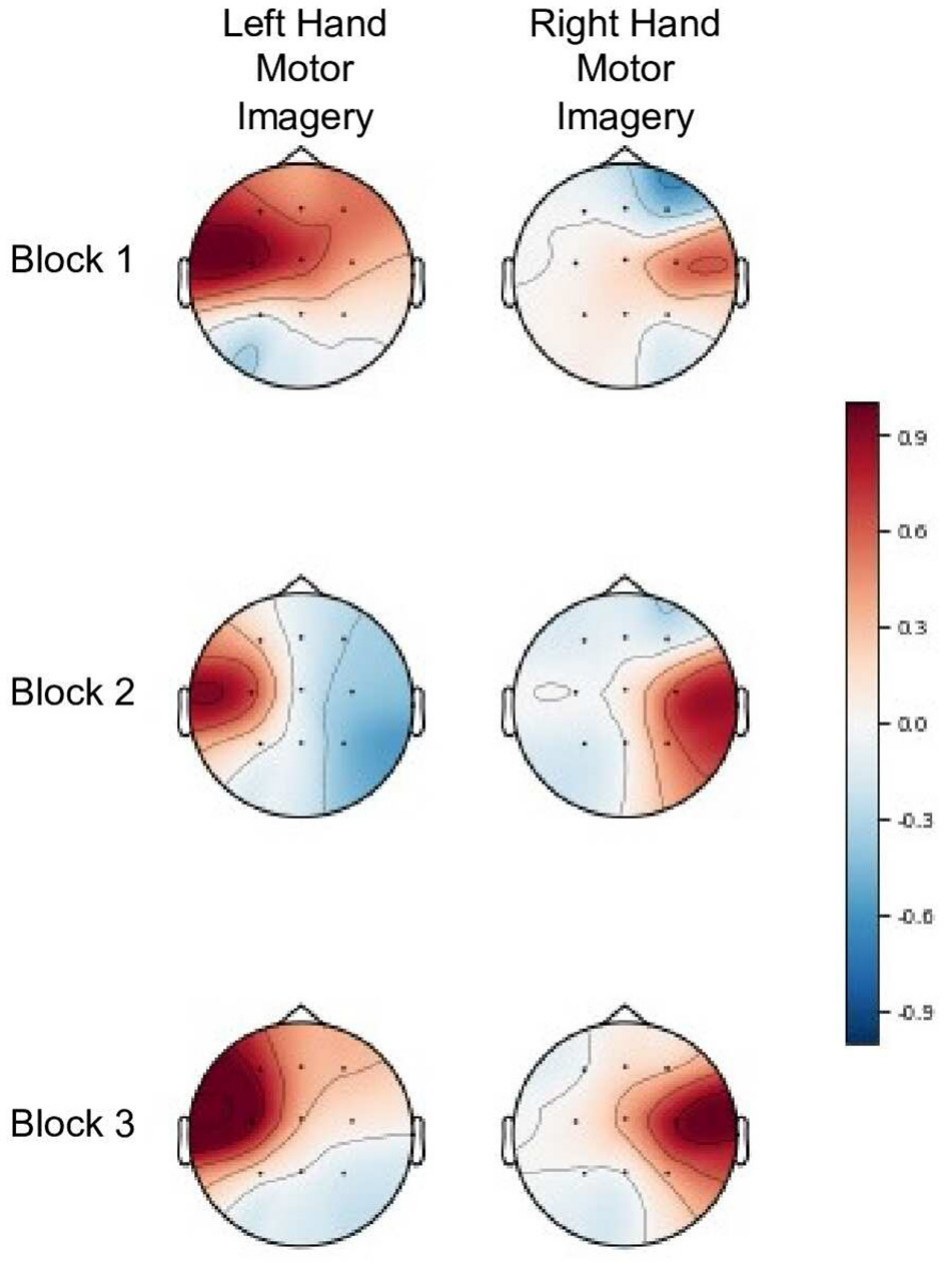
Common spatial patterns (Haufe et al., 2014) for both left and right hand motor imagery tasks across three blocks for participant 15 from the real EEG-BCI dataset. The *classDistinct* metric for blocks 1–3 was 0.327, 0.423, and 0.454 respectively. The gradual performance improvement in the metric is reflected in the increasing difference between the amplitudes of the unsuppressed ipsilateral SMR and suppressed contralateral SMR (event related desynchronization) in each successive blocks. Patterns are shown in arbitrary units, scaled such that the largest absolute value is 1.

Both the sliding window and weighted *classDistinct* reinforcement metrics achieved favorable results with simulated and real data. With the simulated data, the weighted variant performed slightly better with higher correlation and sign discrimination of the block-wise change. This superiority is, as discussed above, partly due to the tendency of the sliding window's intra-class variation estimate to increase. However, the damping effect of the weighted average method rendered it less susceptible to spurious variations during early stages of the blocks and more likely to respond to sustained changes to inter-class dispersion. Conversely, the lack of a damping effect may explain the sliding window reinforcement sums' higher correlation with block-wise *classDistinct* changes for real EEG data. As the block-wise changes were relatively muted compared to the simulated data (e.g., Figure 6), the damping effect could have contributed to less sensitivity to smaller magnitude changes in performance.

### 4.3. Limitations and additional considerations

While the Riemannian geometry-based metrics provided interpretable changes in performance, their absolute values are not meaningful (Lotte and Jeunet, 2018). Nonetheless, providing learners with an indication of the target level of performance and the gap to target is critical effective learning (Hattie and Timperley, 2007; Lotte et al., 2013). Therefore, it may be beneficial to intermittently supplement trial-wise user-training feedback with classifier-based measures of data separability (e.g., block-wise classifier accuracy) to provide an absolute reference point to users throughout training.

Moreover, while the absolute values of the metrics are not interpretable by users, it is relevant to consider how metric values may be impacted by the number of channels and trial length, which were both fixed in this analysis. Generally, increasing the number of channels will increase the Riemannian distance between trial covariance matrices (Congedo et al., 2017). However, the impact of the number of channels on the *classDistinct* and *classStability* metrics would be heavily influenced by the channel locations and the mental tasks employed. For example, if the electrical activity captured by an additional channel is similar (different) for all mental tasks, then inclusion of that channel would likely reduce (increase) the difference between class means and dampen (amplify) relative changes in the metrics.

Similarly, the influence of the trial length would be dependent upon the mental task and the variation in the signal covariance matrix throughout the trial. Generally, however, longer trials would result in lower relative metric changes as the covariance estimates, benefiting from the additional samples, would be more stable and less sensitive to short term signal anomalies. Conversely, if trials are excessively long, there is risk that users fail to sustain the neural modulation for the entirety of the trial. The covariance estimates and metrics would then less accurately reflect EEG signal properties associated with the mental tasks, thereby artificially reducing the value of the metrics. It would be recommended, therefore, when utilizing these metrics to judiciously determine a trial length that is sufficiently long to mitigate sensitivity to short term anomalies and sufficiently short to minimize the influence of superfluous non-task related data.

Additionally, the reinforcement sums calculated here were purely theoretical and only their numerical values were considered during analysis. In real training scenarios, variation in user interpretation of feedback would influence the effectiveness of the reinforcement sums at guiding training. Furthermore, in scenarios such as motor imagery where the control tasks have expected and stereotyped patterns, it may be prudent to review common spatial pattern visualizations (Haufe et al., 2014) after each block to confirm whether the numerical metric changes have physiologically plausible interpretations (as in the example in Figure 6). Nonetheless, our findings encourage future exploration of effective presentations of these numerical metrics as feedback and their impact on user learning during BCI training.

## 5. Conclusion

Motivated by the persistent challenge of BCI inefficiency, we introduced and evaluated variants of Riemannian geometry-based metrics of SMR-BCI user performance. The adapted metrics were designed in conformity to guidelines from skill acquisition literature and instructional design. In analyses of simulation and real SMR-BCI data, we found that our proposed weighted and sliding window *classDistinct* and *classStability* trial-wise reinforcement metrics outperformed classifier-based and running *classDistinct*/*classStability* metrics in accurately reflecting block-wise trends in user performance changes. Future studies should investigate how to effectively present these performance metrics as feedback to users and assess whether such feedback can improve BCI-user learning rates.

## Data availability statement

The raw data supporting the conclusions of this article will be made available by the authors, without undue reservation.

## Author contributions

NI conceptualized and designed the study, generated the simulated data, conducted analyses, and drafted and revised the initial manuscript. TC oversaw study design, data analyses, and reviewed and revised the manuscript. Both authors contributed to the article and approved the submitted version.
