# Corrigendum: Riemannian geometry-based metrics to measure and reinforce user performance changes during brain-computer interface user training

**DOI:** 10.3389/fncom.2023.1286681

**Published:** 2023-11-17

**Authors:** Nicolas Ivanov, Tom Chau

**Affiliations:** ^1^PRISM Lab, Bloorview Research Institute, Holland Bloorview Kids Rehabilitation Hospital, Toronto, ON, Canada; ^2^Institute of Biomedical Engineering, University of Toronto, Toronto, ON, Canada

**Keywords:** brain-computer interface (BCI), electroencephalography (EEG), user training, Riemannian geometry, user evaluation, simulation

In the published article, there was an error. An additional detail regarding the computation of the weighted average *classDistinct* and *classStability* metrics was missing.

A correction has been made to Section 2. Materials and methods, subsection “*2.1 Performance metric design*”, paragraph 7. This sentence previously stated:

“The intra-class dispersion is computed using:


(6) $$\begin{array}{c} \left(1-\alpha_{2}\right)\Phi_{k-1,c}+\alpha_{2}\phi_{k,c} \end{array}$$

where α_2_ ∈ [0, 1] is a constant, *ϕ*_*k*−1, *c*_ is the intra-class dispersion for the class *c* trials of the (*k*-1)th block, and *ϕ*_*k, c*_ is the intra-class dispersion of class *c* trials computed only during the current (*k*th) block.”

The corrected sentences appear below:

“For the weighted average *classDistinct* and *classStability* metrics, we made the following modification to the calculation of the intra-class dispersion. We split the set of trials, *T*, into *N*_*s*_ subsets of *N*_*t*_ trials, *T*_*j*_, such that


$$T_{1}\cup T_{2}\cup \cdots \cup T_{N_{s}}=T.$$

Subsets were formed by splitting trials according to the chronological order in which they were performed; for example, the first *N*_*t*_ trials performed during a block would be grouped into subset *T*_1_. Using these subsets, we computed a modified intra-class dispersion as:


$$\begin{array}{l} \Phi^{*}=\frac{1}{N_{s}}\frac{1}{N_{t}}\overset{N_{s}}{\underset{j=1}{\sum }}\overset{N_{t}}{\underset{i=1}{\sum }}\delta_{R}\left({\bar{\Gamma }}_{T_{j}},\;\Gamma_{T_{j,i}\;}\right)\; \end{array}$$

where *N*_*s*_ is the number of trial subsets, *N*_*t*_ is the number of trials in each subset, ${\bar{\Gamma }}_{T_{j}}$ is the mean covariance matrix of trials within the *j*^*th*^ subset of trials, Γ_*T*_*j, i*__ is the covariance matrix of the *i*^*th*^ trial within subset *T*_*j*_, and δ_*R*_ denotes the Riemannian distance. The motivation behind this modification was to reduce the impact of signal non-stationarities that may artificially increase the intra-class dispersion when considering a large number of trials. For our analysis, we set *N*_*t*_ = 5. Trial subsets were disjoint save for when computing within-block post-trial intra-class dispersion values. If the number of trials completed within the block was not divisible by *N*_*t*_, subset *T*_*N*_*s*__ was formed using the most recently completed *N*_*t*_ trials; consequently, this subset could share up to *N*_*t*_−1 trials with subset *T*_*N*_*s*_ − 1_.

The post-trial intra-class dispersion was computed using this modified intra-class dispersion:


(6) $$\begin{array}{c} \left(1-\alpha_{2}\right)\Phi_{k-1,c}^{*}+\alpha_{2}\phi_{k,c}^{*} \end{array}$$

where α_2_ ∈ [0, 1] is a constant, $\Phi_{k-1,c}^{*}$ is the modified intra-class dispersion for the class *c* trials of the (*k*−1)^*th*^ block, and $\phi_{k,c}^{*}$ is the modified intra-class dispersion of class *c* trials completed only during the current (*k*th) block.”

The authors apologize for this error and state that this does not change the scientific conclusions of the article in any way. The original article has been updated.

